# Characteristics of gut microbiota in children with language developmental delay

**DOI:** 10.3389/fped.2026.1859609

**Published:** 2026-09-03

**Authors:** Yawen Li, Shouyi Wang, Huiping Xu, Xuan Nie, Dongchi Zhao, Wenkai Zhu, Rui Zhu

**Affiliations:** 1 Department of Pediatrics, Zhongnan Hospital of Wuhan University, Wuhan, Hubei, China; 2 Department of Kinesiology, Université de Montréal, Montréal, QC, Canada

**Keywords:** 16S rDNA, characteristics, gut microbiota metabolites, language developmental delay, Sphingobacteriia

## Abstract

**Objective:**

To explore the characteristics of gut microbiota in children with Language Developmental Delay (LDD).

**Methods:**

Children aged 0–6 years diagnosed with LDD at the Pediatric Outpatient Department of Zhongnan Hospital of Wuhan University, and healthy children undergoing physical examinations during the same period (June 2023 to June 2024) were enrolled as study subjects. Fecal samples were collected, and the characteristics of gut microbiota were assessed using 16S rDNA high-throughput sequencing.

**Results:**

A total of 50 children were enrolled, including 24 in the LDD group average age (4.1 ± 1.7) years and 26 in the control group average age (4.0 ± 1.5) years, with no significant demographic differences (*P* > 0.05). The Gesell developmental score in the LDD group (60 ± 12) was significantly lower than that in the control group (102 ± 15) (*P* < 0.05). At the phylum level, the dominant phyla were Firmicutes, Bacteroidetes, Actinobacteria, and Proteobacteria, with no significant difference in abundance between groups (*P* > 0.05). Alpha diversity indices, including Observed species, Chao1 index, Shannon index, and Simpson index, showed no significant difference (*P* > 0.05). Beta diversity analysis (PCoA) revealed no significant difference in microbial community structure between the two groups. Linear discriminant analysis Effect Size (LEfSe) analysis (LDA score > 2) indicated that the LDD group lacked certain bacterial taxa compared to the control group, including Mycobacteriaceae, Sphingobacteriaceae, Sphingobacteriales, Sphingobacteriia, Caulobacteriaceae, Caulobacterales, Erythrobacteraceae, Sphingomonadaceae, and Sphingomonadales.

**Conclusion:**

Children with Language Developmental Delay exhibited gut microbial dysbiosis, but no specific gut microbial composition was identified.

## 1 Introduction

Language Developmental Delay (LDD) refers to a condition in which a child's language development follows the normal sequence but progresses at a slower rate than that of typically developing children, failing to reach the level expected for their age (1). The prevalence of LDD is relatively high in preschool children, reported as 3%–7% internationally and up to 15% in 2-year-old children in China (2). Recent studies (3–5) suggest that LDD is associated with multiple factors, including congenital factors, environmental influences, and dietary/behavioral issues. Concurrently, emerging evidence indicates that gut microbiota diversity can impact neurocognitive development in children, as seen in disorders like Autism Spectrum Disorder (ASD) and Attention Deficit Hyperactivity Disorder (ADHD), which are now considered within the scope of gut microbiota-targeted therapies (6–9). However, LDD, as another neurocognitive developmental disorder, has not received sufficient attention in this regard. Currently, there is a lack of literature reporting on the characteristics of gut microbiota in children with LDD. Therefore, this study aimed to explore the gut microbiota features in children with LDD to provide a reference for research on gut microecology in this population.

## 2 Subjects

### 2.1 Subjects

Children aged 0–6 years diagnosed with LDD at the Pediatric Outpatient Department of Zhongnan Hospital of Wuhan University, and healthy children undergoing physical examinations from June 2023 to June 2024 were enrolled. Inclusion criteria: Completion of the Gesell Developmental Diagnosis Scale. Specifically for the LDD group: Age 0–6 years and Gesell language development score < 76. Exclusion criteria: 1) Hearing impairment; 2) Oral malformations; 3) Diagnosis of Autism Spectrum Disorder; 4) History of acute or chronic infectious diseases within the past month; 5) Use of antimicrobial agents or probiotics within the past three months. Control group inclusion criteria: No use of antimicrobial agents or probiotics within the past three months, no neurological or psychological behavioral abnormalities, and no illness or antibiotic use within the past three months. Fecal samples were collected, and gut microbiota diversity and dominant flora were assessed via 16S rDNA high-throughput sequencing. This study was approved by the Ethics Review Committee of Zhongnan Hospital, Wuhan University (Approval No. 2023048 K). Informed consent was obtained from the parents of all participants.

## 3 Methods

### 3.1 Collection of basic information

The medical records of the children were collected from the hospital computer system of Zhongnan Hospital of Wuhan University, including name, ID number, gender, age, previous diseases and medication, Gesell scale score, and date of fecal sample collection.

### 3.2 Fecal sample collection

Fecal samples (approximately 100–1,000 mg) were collected from participants using sterile fecal collection kits (Xiamen ZB Biotech, Lot No. 93050W). Samples were were stored at room temperature in the kit's preservation solution, and were processed within 7 days. Operators wore sterile gloves and masks. Parents completed a detailed basic information questionnaire.

### 3.3 Fecal DNA extraction

DNA Extraction: Microbial DNA was extracted using the QIAsymphony DSP Virus/Pathogen Midi Kit (Qiagen, Cat. No. 937055), following the manufacturer's protocol. 16S rDNA Amplification: Th hypervariable region was amplified using the Ion 16S Metagenomics Kit (Thermo Fisher Scientific, Cat. No. A26216). Sequencing: High-throughput sequencing was performed on the Illumina NovaSeq platform (paired-end, 2 × 250 bp).

### 3.4 Bioinformatics analysis

Quality Control: Raw sequencing data were filtered (Q-score ≥ 20) and chimera-checked using USEARCH (Tiburon, CA, USA) against the SILVA database (Braunschweig, Germany). OTU Clustering: Operational taxonomic units (OTUs) were defined at 97% sequence similarity. Taxonomic Annotation: OTUs were classified using the RDP Classifier (East Lansing, MI, USA). Diversity Analysis: α-Diversity: Calculated using Mothur Ann Arbor, MI, USA). β-Diversity: Principal coordinates analysis (PCoA) based on UniFrac distances. Differential Abundance: Linear discriminant analysis Effect Size (LEfSe, Boston, MA, USA) analysis Linear Discriminant Analysis (LDA) score > 2.0 identified significantly enriched taxa.

### 3.5 Statistical analysis

Data were analyzed using SPSS 26.0. Normally distributed measurement data are expressed as mean ± standard deviation (x¯ ± s). Non-normally distributed measurement data are expressed as median (P25, P75). Count data are expressed as *n* (%). The inter-group comparison of the Gesell score and the age of children were conducted using the t-test, the comparison of gender was performed using the chi-square test, the richness of the microbial richness at the phylum level was evaluated using the Mann–Whitney U test, and the comparison of α diversity was carried out using the Wilcoxon test. A *P*-value < 0.05 was considered statistically significant.

## 4 Results

### 4.1 General clinical data

Fifty children were enrolled: 24 in the LDD group [14 males, 10 females, average age (4.1 ± 1.7) years] and 26 in the control group [14 males, 12 females, average age (4.0 ± 1.5) years]. There were no significant differences between the basic Information (*P* > 0.05). The Gesell score was significantly lower in the LDD group (60 ± 12) than in the control group (102 ± 15) (*P* < 0.05) Table 1.

**Table 1:** Basic information of case and control group.

Class		Case	Control	*P*
sex	total	24	26	0.72
	male	14	14	
	famale	10	12	
age		(4.1 ± 1.7)	(4.0 ± 1.5)	0.85
Gesell Score		60 ± 12	102 ± 15	0.02

## 5 Gut Microbiota analysis results

### 5.1 Species accumulation curve and heatmap analysis

**Figure 1:**
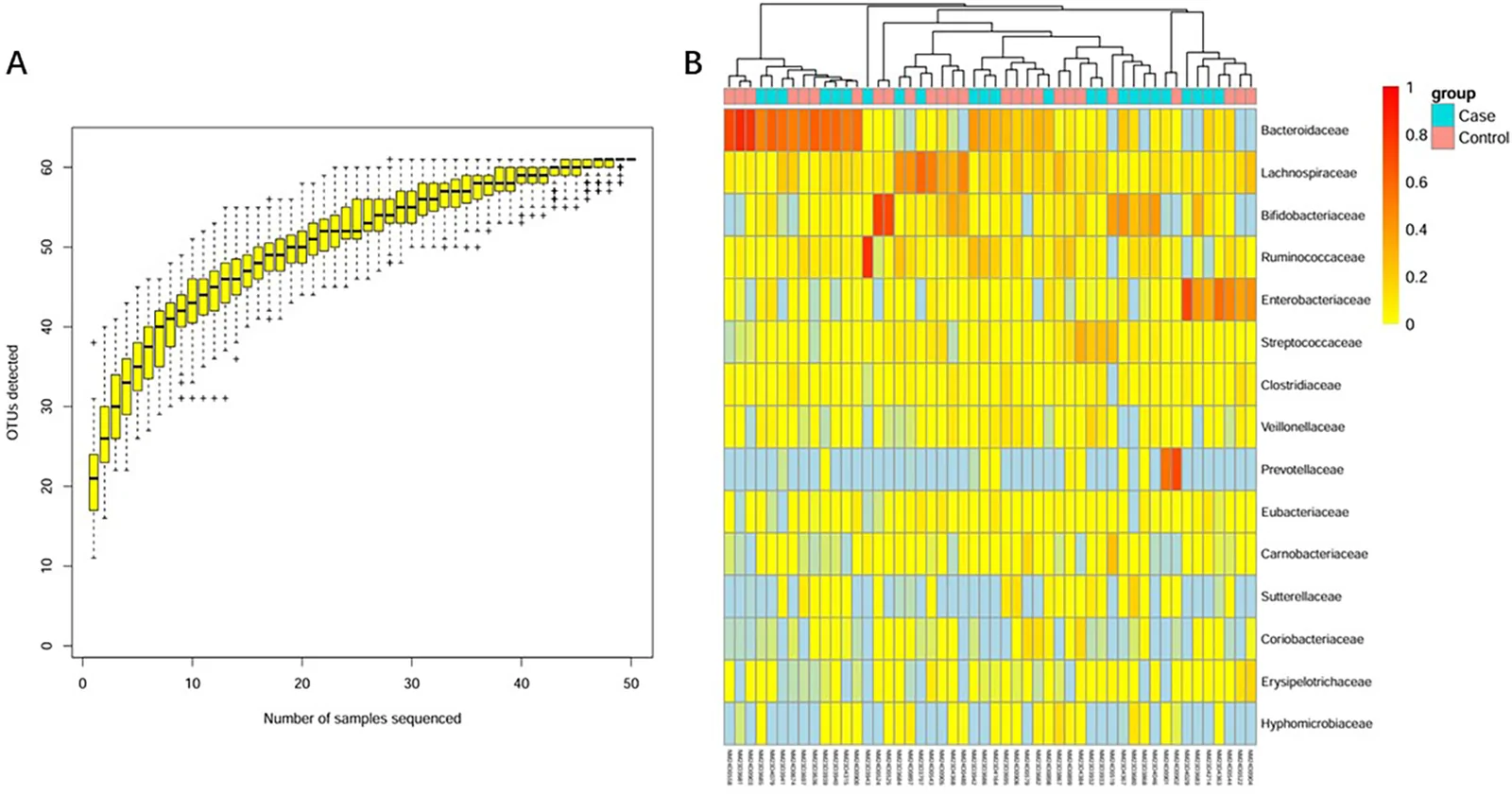
Species accumulation curve and overlapped species richness heatmap between two groups. **(A)** The species accumulation curves. The samples were drawn and processed, with the horizontal coordinate representing the number of samples and the vertical coordinate representing the number of OTUs. **(B)** The heatmap of species richness of samples in genus level. The vertical clustering represents the similarity of the abundance of the species in each sample. The species richness between two groups overlapped significantly.

The number of reads corresponding to different samples varies greatly. To avoid the bias caused by the different sizes of sequencing data of samples, we randomly sampled each sample to the same depth when the sequencing depth of each sample was sufficient. At the phylum level, nine phyla were identified. Firmicutes was the most dominant. The dominant phyla for all subjects were Firmicutes, Bacteroidetes, Actinobacteria, and Proteobacteria. Others included Cyanobacteria, Fusobacteria, and Verrucomicrobia. The LDD group showed very low abundance of Synergistetes and Tenericutes. No significant difference was found in the relative abundance of dominant phyla between groups (*P* > 0.05). Table 2, Figure 1B showed the top eight genera were *Bacteroides*, *Bifidobacterium*, *Ruminococcus*, *Streptococcus*, *Clostridium*, *Faecalibacterium*, *Eubacterium*, and *Shigella*. The species richness between two groups overlapped significantly.

**Table 2:** Comparison of microbial richness at the phylum level between the case group and the normal control group.

OTU ID	Case	Control	P
Actinobacteria	0.0523（0.0215，0.1343）	0.0918（0.0055，0.2179）	0.801
Bacteroidetes	0.1934（0.0476，0.5597）	0.1691（0.0020，0.5695）	0.683
Cyanobacteria	0.0000（0.0000，0.0000）	0.0000（0.0000，0.0000）	0.604
Firmicutes	0.0347（0.2716，0.6167）	0.4992（0.2508，0.6238）	0.741
Fusobacteria	0.0000（0.0000，0.0004）	0.0000（0.0000，0.0000）	0.469
Proteobacteria	0.0798（0.0474，0.2051）	0.0528（0.0187，0.1334）	0.077
Synergistetes	0.0000（0.0000，0.0000）	0.0000（0.0000，0.0000）	0.089
Tenericutes	0.0000（0.0000，0.0000）	0.0000（0.0000，0.0000）	0.337
Verrucomicrobia	0.0000（0.0000，0.0000）	0.0000（0.0000，0.0000）	0.891

OUT ID, operational taxonomic unit identification.

### 5.2 Alpha diversity analysis

**Figure 2:**
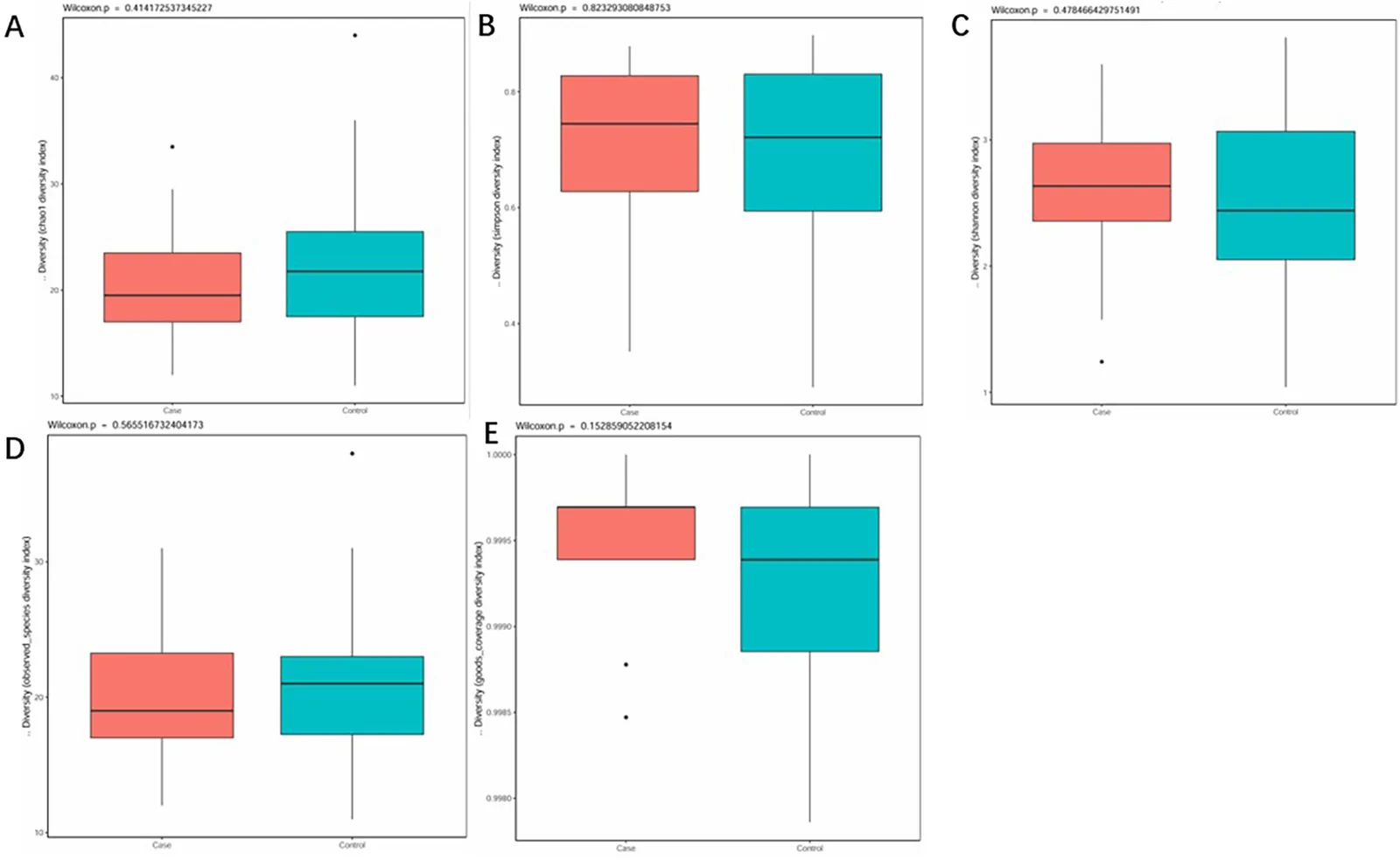
α-diversity of gut microbiota in children with LDD and control group shown no difference. The abscissa represents grouping and the ordinate represents *α*-diversity indices. **(A)** Chao1 index (*P* = 0.414) **(B)** Simpson index (*P* = 0.823) **(C)** Shannon index (*P* = 0.473) **(D)** Observed species (*P* = 0.565) **(E)** goods coverage(*P* = 0.153). The Wilcoxon test was used for the comparison between groups, and no significant statistical differences were observed in any *α*-diversity indices (*P* > 0.05).

To further elucidate the characteristic differences in gut microbiota between children in the case and control groups, we conducted comprehensive analyses of microbial diversity based on species annotation and community composition. All the data were used for analysis. No statistically significant differences were found between groups for any *α*-diversity indices (*P*＞ 0.05) (Figure 2).

### 5.3 Beta diversity analysis

**Figure 3:**
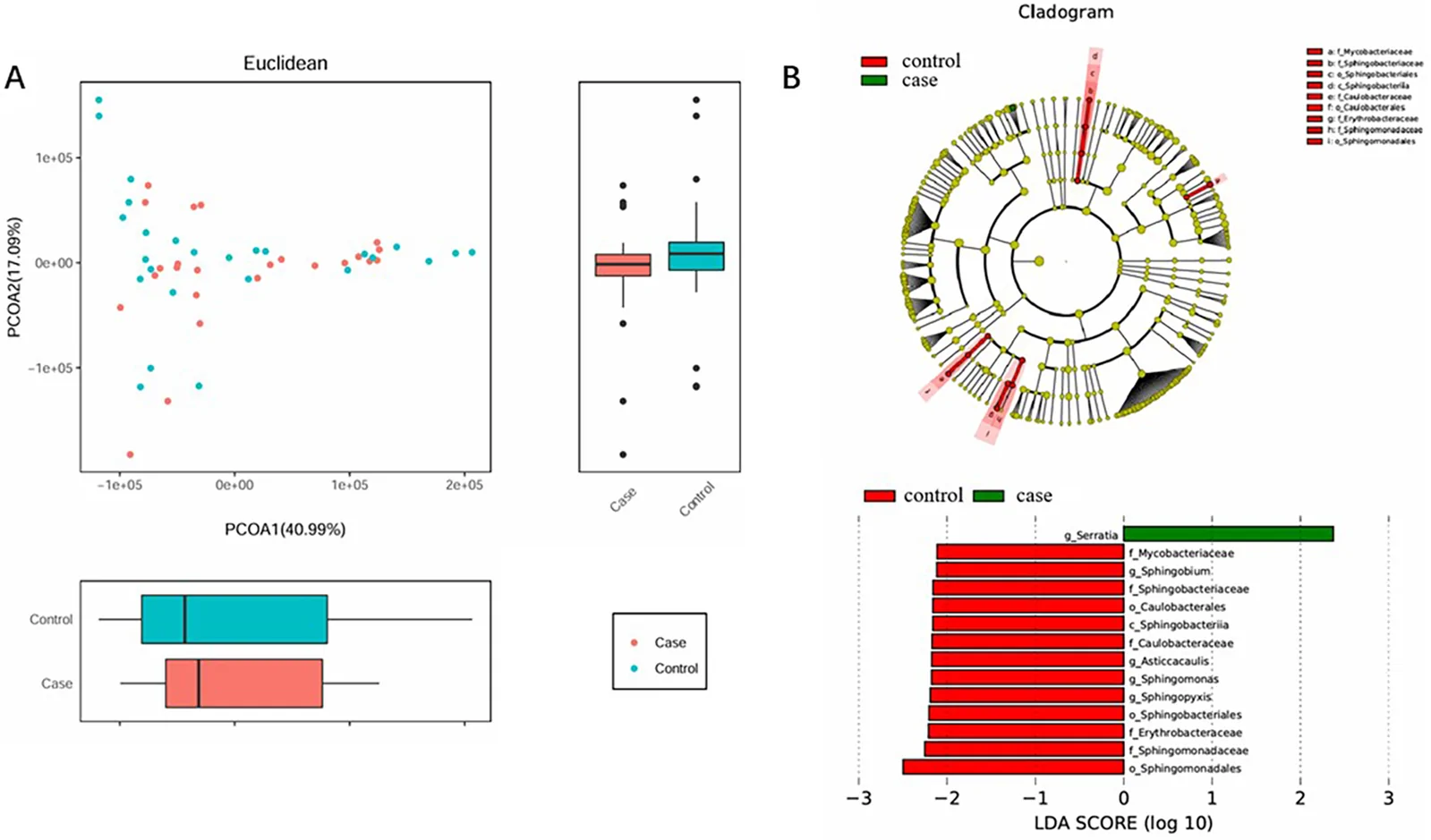
There was no difference of *β*-diversity of gut microbiota between children with LDD and control group, but dominant bacteria were identified by LEfSe analysis. **(A)** The β diversity was analyzed using Euclidean method.PCoA2 was on the right and PCoA1 below, which overlapped significantly. PCoA analysis based on UniFrac distances showed no significant separation between groups, with principal coordinates explaining 40.99% of variance. The ADONIS analysis result indicated that the R^2^ values was 0.0092 and the *P* value was 0.89, suggesting no significant statistical difference of *β* diversity between two groups. **(B)** Through LEfSe analysis, it was found that when the LDA score was greater than 2, the bacterial communities of the normal control group had an absolute dominant strain starting from the phylum level, which was different from that of the case group. And it also revealed that the LDD group had significantly enriched bacteria like Serratia compared to the control group, which could be harmful.

PCoA analysis based on UniFrac distances showed no significant separation between groups (Figure 3A), with principal coordinates explaining 40.99% of variance. All the data were used for analysis. The ADONIS analysis results indicated that the R^2^ values was 0.0092 and the *P* value was 0.89, suggesting no significant statistical difference of β diversity between two groups.

### 5.4 LEfSe analysis

When LDA score > 2, LEfSe analysis revealed that the control group had significantly enriched bacterial taxa compared to the LDD group, including Mycobacteriaceae, Sphingobacteriaceae, Sphingobacteriales, Sphingobacteriia, Caulobacteriaceae, Caulobacterales, Erythrobacteraceae, Sphingomonadaceae, and Sphingomonadales (Figure 3B).

## 6 Discussion

Intestinal microbiota are normal microorganisms residing on the intestinal mucosal surface, capable of producing small organic compounds called N-acyl amides that interact with receptors to regulate physiological health, participating in various signaling pathways including immunity, behavior, and metabolism (10). The complex interplay between intestinal microbiota and the host constitutes the gut microecosystem, the largest microbial ecosystem in the human body. This system influences the central nervous and intestinal systems through neural, endocrine, immune, and metabolic pathways—a relationship termed the gut-brain-microbiota axis (11). Language development, as a key aspect of neurocognitive function, has garnered increasing attention. The critical period for language development occurs between 18 and 36 months of age (12). Untreated language delays can impair learning abilities and cause long-term deficits (13). A groundbreaking study in 2024 (14) linked the gut microbiota to developmental stuttering for the first time. The researchers used a Gnptab gene mutation mouse model and found that the *α* diversity of the gut microbiota in the case group mice was significantly reduced, and predicted differences in the short-chain fatty acid and lipopolysaccharide synthesis pathways. Even within ASD, whether there is language delay can affect the composition of the microbiota. Language-delayed children with ASD showed a predominance of Clostridium, while language-normal children with ASD were mainly dominated by Bacteroidetes/Prevotella. These studies all suggest that the gut microbiota is related to language development (15). However, as no prior studies have reported on gut microbiota in children with LDD, this study analyzed these characteristics to provide a reference.

Gut microbes are numerous and diverse, crucial for health. Their functions include aiding intestinal barrier establishment and maintenance, stimulating epithelial regeneration via short-chain fatty acid production, promoting mucus secretion, and nourishing the mucosa (16, 17). Growing evidence (18–21) links gut microbiota abnormalities to neurocognitive disorders, but data on LDD is lacking. Gut microbiota establishment primarily occurs within the first three years of life. Initially dominated by Actinobacteria and Proteobacteria, it gradually shifts to Firmicutes and Bacteroidetes, increasing diversity and stabilizing around age 3, coinciding with nervous system maturation. This study found four dominant phyla in children with LDD: Firmicutes, Bacteroidetes, Proteobacteria, and Actinobacteria, alongside Cyanobacteria, Fusobacteria, and Verrucomicrobia, with very low Synergistetes and Tenericutes. No significant difference in abundance was found compared to controls (*P* > 0.05). At the genus level, common genera in children with LDD (*Bacteroides*, *Bifidobacterium*, etc.) showed no significant difference vs. controls (*P* > 0.05), suggesting similar richness. Some ASD studies (22, 23) align with our findings of no difference in these phyla's abundance, while others report differences (24), studies have found that in the ASD group, the phylum Bacteroidetes and Proteobacteria, at the genus level, *Desulfovibrio, Acetanaerobacterium, Parabacteroides, Bacteroides,* and *Desulfovibrio spp.* and *Bacteroides vulgatus*, as well as at the species level, were higher than in the control group. Additionally, at the phylum level, the Actinobacteria and Firmicutes, and at the genus level, *Weissella, Turicibacter, Clostridium, Anaerofilum, Pseudoramibacter, Ruminococcus, Streptococcus, Anaerovorax, Dialister, Lactococcus, Leuconostoc, Ethanoligenens, Helcococcus,* and *Alkaliphilus* were higher in the control group than in the ASD group. A study (25) on young children with ASD and age-matched neurologically healthy control groups indicated that the phylum Bacteroidetes and Proteobacteria in children with ASD were significantly increased, while the phylum Actinobacteria decreased. It is notable that the dominant bacterium in the infant gut microbiota, *Bifidobacterium longum*, significantly decreased, while a late colonizing bacterium and a major butyrate-producing bacterium in a healthy human gut, *Faecalibacterium prausnitzii*, increased in children with ASD. Similarly, a report (26) stated that compared with control children, the genera *Bacteroides, Prevotella, Lachnospiracea_incertae_sedis, Megamonas*, and Bacteroidetes/Firmicutes were increased in autistic children. The difference in the richness of the intestinal microbiota between the two may be related to the fundamental differences between language development delay and ASD. From the clinical manifestations, ASD is a multi-symptom syndrome disorder that includes language development delay, while children with language development delay only have speech development disorders. In terms of immune and metabolism, the continuous abnormal activation of the immune inflammator*y* axis in children with ASD, such as the continuous abnormal high expression of IFN-γ, leads to the enrichment of Veillonellaceae in the intestinal microbiota and the reduction of Bifidobacterium, while accompanied by the reduction of short-chain fatty acids (butyric acid) and the accumulation of neurotoxins such as *p*-cresol/propionic acid (27). However, these differences do not apply to children with language development delay. In children with LDD, the abnormal regulation of mRNA translation mediated by CAPRIN1 directly affects the formation and remodeling of synapses. Abnormal neuronal calcium signaling can directly impair the development of language networks. Neurons are highly sensitive to oxidative damage, and increased oxidative stress may lead to selective vulnerability of language-related brain regions (28). At the genetic level, the latest multi-omics research has revealed an astonishing discovery—many bacteria that do not change in abundance in the intestines of children with autism spectrum disorder have also undergone significant changes in their internal single nucleotide variations and structural variations (29). This may be the fundamental reason for the more widespread microbiota imbalance in children with ASD.

Alpha diversity indices (Observed species, Chao1 index, Shannon index, Simpson index) showed no significant difference between LDD and control groups (*P* > 0.05), suggesting alpha diversity may not be a key factor in LDD. Similar null findings for alpha diversity have been reported in comparisons of typically developing children with those with ADHD or ASD (30, 31). Of course, the presence of the microbial community varies in the gastrointestinal tract, ranging from a few in the stomach and small intestine to a large amount in the colon. Its composition is influenced by various factors, such as environmental factors, the mother's pregnancy status, as well as external and internal factors such as different dietary habits and lifestyles.

LEfSe analysis (LDA > 2) indicated the control group had enriched taxa including Sphingobacteriaceae, Sphingobacteriales, Sphingobacteriia, Sphingomonadaceae, Sphingomonadales, Mycobacteriaceae, Caulobacteriaceae, Caulobacterales, and Erythrobacteraceae. In the case group, the Serratia bacteria are classified as opportunistic pathogens and are obligate anaerobes. Serratia typically causes direct intracranial infections that affect brain function. Additionally, studies have shown that even without infection, the lipopolysaccharide (LPS) on the cell wall of Serratia can directly act on neurons and neuromuscular junctions, altering the efficiency of synaptic transmission, thereby influencing behavioral cognition and sensory responses (32). Notably, children with LDD lacked Sphingobacteriia (within Bacteroidetes). Bacteroidetes are the only known gut commensals producing sphingolipids, which can induce anti-inflammatory responses via the mevalonate pathway, crucial for maintaining gut homeostasis. Sphingolipid metabolites are vital for brain neuron formation and structure (33–36). Tamana et al. (7) conducted a study on 405 infants, they analyzed the microbiota in fecal samples from infants at an average age of 4 and 12 months using 16S rRNA gene sequencing, which found that male infants with a dominant Bacteroides microbiota group had better cognitive and language development. The abundance of Bacteroides genus in the gut microbiota at 2 years of age was positively correlated with cognitive and language scores, indicating a positive correlation between the Bacteroidetes intestinal microbiota in the late infancy and subsequent cognitive and language development. Reduced Bacteroidetes has also been reported in 2–3-year-olds later diagnosed with ASD (36). The lack of Bacteroidetes-related microbes like Sphingobacteriia in children with LDD may also be a contributing pathogenic factor. The Sphingomonadaceae, Caulobacteriaceae and Erythrobacteraceae families in the control group belong to the *α*-proteobacteria class. Studies have shown that these bacterial groups have the potential to produce vitamin B12, especially the Sphingomonadaceae family (37). Vitamin B12 plays a crucial role in neural cognitive functions by maintaining myelin integrity, participating in methylation reactions, regulating homocysteine metabolism and ensuring the energy supply of nerve cells (38) Research has found that in the infant's intestinal tract, the biological synthesis function of vitamin B12 is significantly positively correlated with all developmental skills of children (cognition, language, movement and stress regulation ability). After vitamin B12 treatment for infants with vitamin B12 deficiency, significant myelin repair and axon recovery were observed in multiple brain regions, and the improvement degree of these regions was positively correlated with the improvement of the infant's neurological development, especially language development scores (39, 40). Therefore, we suspect that the lack of the *α*-proteobacteria class, especially the Sphingomonadaceae family, in the case group may cause disease by affecting the vitamin B12 level in children with delayed language development. Future studies can further detect the vitamin B12 level in the peripheral blood of children with LDD to verify this conjecture. Perhaps vitamin B12 supplementation can be used for treatment intervention. Further research is needed.

### 6.1 Study limitations

The sample size was limited, and participants were aged 0–6 years. The study focused on Chinese children, racial and environmental differences compared to other populations may influence results. Future studies with larger, more diverse samples are needed to better characterize gut microecology in LDD.

## 7 Conclusion

Children with Language Developmental Delay exhibited gut microbial dysbiosis. While overall gut microbiota richness and diversity showed no significant difference compared to healthy controls. The deficiency of specific Bacteroidetes-related bacteria, such as Sphingobacteriia, The α-proteobacteria group, such as the Sphingomonadaceae in children with LDD may be a potential pathogenic factor, warranting further investigation.

## Data Availability

The original contributions presented in the study are publicly available. This data can be found here: https://www.ncbi.nlm.nih.gov/bioproject/PRJNA1502771, Accession:PRJNA1502771.

## References

[1] Zhao Z Li Y Zhang W. Progress in language development delay in children. Chin Sci J Hear Speech Rehabil Sci. (2025) 23(2):128–32,168. 10.3969/j.issn.1672-4933.2025.02.005

[2] Nudel R Christensen RV Kalnak N Schwinn M Banasik K Dinh KM. Developmental language disorder—a comprehensive study of more than 46,000 individuals. Psychiatry Res. (2023) 323:115171. 10.1016/j.psychres.2023.115171 36963307

[3] Wu S Zhao J de Villiers J Liu XL Rolfhus E Sun X. Prevalence, co-occurring difficulties, and risk factors of developmental language disorder: first evidence for Mandarin-speaking children in a population-based study. Lancet Reg Health West Pac. (2023) 34:100713. 10.1016/j.lanwpc.2023.100713 37283967 PMC10240373

[4] Matsumoto M Ferreira HA Araujo IB Cardilli-Dias D Molini-Avejonas DR. Correlation between risk factors that influence the development of children's Language. Codas. (2025) 37:e20240131. 10.1590/2317-1782/e20240131en 39879429 PMC11781358

[5] Dilbaz-Gürsoy M Noyan-Erbaş A Esen H Köse A Özcebe E. What influences parenting stress? Examining parenting stress and self-efficacy across groups of children with autism spectrum disorder, at risk of developmental language disorder, and with typically developing language. J Speech Lang Hear Res. (2025) 68:2837–50. 10.1044/2025_JSLHR-24-00672 40324152

[6] van de Wouw M Wang Y Workentine ML Vaghef-Mehrabani E Dewey D Reimer RA. Associations between the gut microbiota and internalizing behaviors in preschool children. Psychosom Med. (2022) 84:159–69. 10.1097/PSY.0000000000001026 34654024

[7] Tamana SK Tun HM Konya T Chari RS Field CJ Guttman DS. Bacteroides-dominant gut microbiome of late infancy is associated with enhanced neurodevelopment. Gut Microbes. (2021) 13:1–17. 10.1080/19490976.2021.1930875 34132157 PMC8210878

[8] McMath AL Aguilar-Lopez M Cannavale CN Khan NA Donovan SM. A systematic review on the impact of gastrointestinal microbiota composition and function on cognition in healthy infants and children. Front Neurosci. (2023) 17:1171970. 10.3389/fnins.2023.1171970 37389363 PMC10306408

[9] Anaclerio F Minelli M Antonucci I Gatta V Stuppia L. Microbiota and autism: a review on oral and gut microbiome analysis through 16S rRNA sequencing. Biomedicines. (2024) 12:2686. 10.3390/biomedicines12122686 39767593 PMC11726726

[10] Drzazga A Bernat P Nowak A Szustak M Korkus E Gendaszewska-Darmach E. N-acyl glycines produced by commensal bacteria potentiate GLP-1 secretion as GPCR ligands. Biomed Pharmacother. (2024) 180:117467. 10.1016/j.biopha.2024.117467 39362066

[11] Cerdó T Nieto-Ruíz A García-Santos JA Rodríguez-Pöhnlein A García-Ricobaraza M Suárez A. Current knowledge about the impact of maternal and infant nutrition on the development of the microbiota-gut-brain axis. Annu Rev Nutr. (2023) 43:251–78. 10.1146/annurev-nutr-061021-025355 37603431

[12] Patel RA Panche AN Harke SN. Gut microbiome-gut brain axis-depression: interconnection. World J Biol Psychiatry. (2025) 26:1–36. 10.1080/15622975.2024.2436854 39713871

[13] Kasari C Shire S Shih W Landa R Levato L Smith T. Spoken language outcomes in limited language preschoolers with autism and global developmental delay: rCT of early intervention approaches. Autism Res. (2023) 16:1236–46. 10.1002/aur.2932 37070270 PMC10460274

[14] Nanda S Lamot B Guarino N Usler E Chugani DC Dutta A. Atypical gut microbiota composition in a mouse model of developmental stuttering. Sci Rep. (2024) 14:23457. 10.1038/s41598-024-74766-x 39379558 PMC11461706

[15] Zhang DQ. Analysis of the influence of interaction between intestinal flora and neurophysiological factors on language development in children with autis. Chin J Hear Speech Rehabil Sci. (2025)23(5):542–547. 10.3969/j.issn.1672-4933.2025.05.022

[16] Ullah H Arbab S Tian Y Liu CQ Chen Y Qijie L. The gut microbiota-brain axis in neurological disorder. Front Neurosci. (2023) 17:1225875. 10.3389/fnins.2023.1225875 37600019 PMC10436500

[17] Long J Chen J Huang H Liang J Pang L Yang K. The associations between gut microbiota and fecal metabolites with intelligence quotient in preschoolers. BMC Microbiol. (2024) 24:431. 10.1186/s12866-024-03579-9 39455934 PMC11515365

[18] Lapidot Y Maya M Reshef L Cohen D Ornoy A Gophna U. Relationships of the gut microbiome with cognitive development among healthy school-age children. Front Pediatr. (2023) 11:1198792. 10.3389/fped.2023.1198792 37274812 PMC10235814

[19] Park JC Sim MA Lee C Park HE Lee J Choi SY. Gut microbiota and brain-resident CD4(+) T cells shape behavioral outcomes in autism spectrum disorder. Nat Commun. (2025) 16:6422. 10.1038/s41467-025-61544-0 40645945 PMC12254219

[20] Su Q Wong O Lu W Wan Y Zhang L Xu W. Multikingdom and functional gut microbiota markers for autism spectrum disorder. Nat Microbiol. (2024) 9:2344–55. 10.1038/s41564-024-01739-1 38977906

[21] Caputi V Hill L Figueiredo M Popov J Hartung E Margolis KG. Functional contribution of the intestinal microbiome in autism spectrum disorder, attention deficit hyperactivity disorder, and rett syndrome: a systematic review of pediatric and adult studies. Front Neurosci. (2024) 18:1341656. 10.3389/fnins.2024.1341656 38516317 PMC10954784

[22] Novau-Ferré N Papandreou C Rojo-Marticella M Canals-Sans J Bulló M. Gut microbiome differences in children with attention deficit hyperactivity disorder and autism Spectrum disorder and effects of probiotic supplementation: a randomized controlled trial. Res Dev Disabil. (2025) 161:105003. 10.1016/j.ridd.2025.105003 40184961

[23] Zeng Q Hu Y Xie L Zhang X Huang Y Ye J. Gut microbiota diversity and composition in children with autism spectrum disorder: associations with symptom severity. Peerj. (2025) 13: e19528. 10.7717/peerj.19528 40492208 PMC12147763

[24] Martínez-González AE Andreo-Martínez P. The role of gut microbiota in gastrointestinal symptoms of children with ASD. Medicina (Kaunas). (2019) 55:408. 10.3390/medicina55080408 31357482 PMC6722942

[25] Liu J Gao Z Liu C Liu T Gao J Cai Y. Alteration of gut microbiota: new strategy for treating autism spectrum disorder. Front Cell Dev Biol. (2022) 10:792490. 10.3389/fcell.2022.792490 35309933 PMC8929512

[26] Zou R Xu F Wang Y Duan M Guo M Zhang Q. Changes in the gut microbiota of children with autism spectrum disorder. Autism Res. (2020) 13:1614–25. 10.1002/aur.2358 32830918

[27] Xu XJ Lang JD Yang J Long B Liu XD Zeng XF. Differences of gut microbiota and behavioral symptoms between two subgroups of autistic children based on γδT cells-derived IFN-γ levels: a preliminary study. Front Immunol. (2023) 14: 1100816. 10.3389/fimmu.2023.1100816 36875075 PMC9975759

[28] Pavinato L Delle VA Carli D Ferrero M Carestiato S Howe JL. CAPRIN1 haploinsufficiency causes a neurodevelopmental disorder with language impairment, ADHD and ASD. Brain. (2023) 146:534–48. 10.1093/brain/awac278 35979925 PMC10169411

[29] Chen W Wang X Zhu R Gao W Tao L Yang R. Integrative multi-omics reveals microbial genomic variants driving altered host-microbe interactions in autism spectrum disorder. Cell Rep Med. (2026) 7:102516. 10.1016/j.xcrm.2025.102516 41421350 PMC12866134

[30] Jia W Liu M Zhou Z Li J Wu S Shen X. [Relationship between gut microbiome and neurodevelopment in early life]. Wei Sheng Yan Jiu. (2024) 53:250–6. 10.19813/j.cnki.weishengyanjiu.2024.02.012 38604961

[31] Bundgaard-Nielsen C Lauritsen MB Knudsen JK Rold LS Larsen MH Hindersson P. Children and adolescents with attention deficit hyperactivity disorder and autism spectrum disorder share distinct microbiota compositions. Gut Microbes. (2023) 15:2211923. 10.1080/19490976.2023.2211923 37199526 PMC10197996

[32] Bernard J Greenhalgh A Istas O Marguerite NT Cooper RL. The effect of bacterial endotoxin LPS on serotonergic modulation of glutamatergic synaptic transmission. Biology (Basel). (2020) 9:210. 10.3390/biology9080210 32781679 PMC7463696

[33] Brown EM Temple ER Jeanfavre S Avila-Pacheco J Taylor N Liu K. Bacteroides sphingolipids promote anti-inflammatory responses through the mevalonate pathway. Cell Host Microbe. (2025) 33:901–14. 10.1016/j.chom.2025.05.007 40449488 PMC12169427

[34] Nogay NH Nahikian-Nelms M. Can we reduce autism-related gastrointestinal and behavior problems by gut microbiota based dietary modulation? A review. Nutr Neurosci. (2021) 24:327–38. 10.1080/1028415X.2019.1630894 31216957

[35] van de Wouw M Wang Y Workentine ML Vaghef-Mehrabani E Barth D Mercer EM. Cluster-specific associations between the gut microbiota and behavioral outcomes in preschool-aged children. Microbiome. (2024) 12:60. 10.1186/s40168-024-01773-5 38515179 PMC10956200

[36] Dan Z Mao X Liu Q Guo M Zhuang Y Liu Z. Altered gut microbial profile is associated with abnormal metabolism activity of autism Spectrum disorder. Gut Microbes. (2020) 11:1246–67. 10.1080/19490976.2020.1747329 32312186 PMC7524265

[37] Wijaya SC Richi M Waturangi DE Yulandi A. Linking metagenomic insight to cultivable microbes: isolation of a vitamin B(12)-producing sphingomonad from Indonesian tempeh. BMC Microbiol. (2026) 26:127. 10.1186/s12866-025-04681-2 41572158 PMC12911007

[38] Larvie DY Güitrón LC Venkatramanan S Palma MX Steel LB Kuriyan R. Vitamin B12 supplementation for growth, development, and cognition in children. Cochrane Database Syst Rev. (2026) 6:D15264. 10.1002/14651858.CD015264.pub2 PMC13227581 42227307

[39] Oliphant K Cruz AW Ilyumzhinova R Mbayiwa K Sroka A Xie B. Microbiome function and neurodevelopment in black infants: vitamin B (12) emerges as a key factor. Gut Microbes. (2024) 16:2298697. 10.1080/19490976.2023.2298697 38303501 PMC10841033

[40] Vyas S Kumar P Salan T Sankhyan N Dash S Srikumar S. Characterization of brain microstructural changes in children with infantile vitamin B12 deficiency using diffusion tensor imaging. Neuroradiology. (2025) 67:3349–58. 10.1007/s00234-025-03831-7 41174081

